# Supplementary figures and images for: Host cystathionine-γ lyase derived hydrogen sulfide protects against Pseudomonas aeruginosa sepsis
Source: PLoS Pathog. 2021 Mar 26;17(3):e1009473. doi: 10.1371/journal.ppat.1009473 (PMC8051778; doi:10.1371/journal.ppat.1009473)

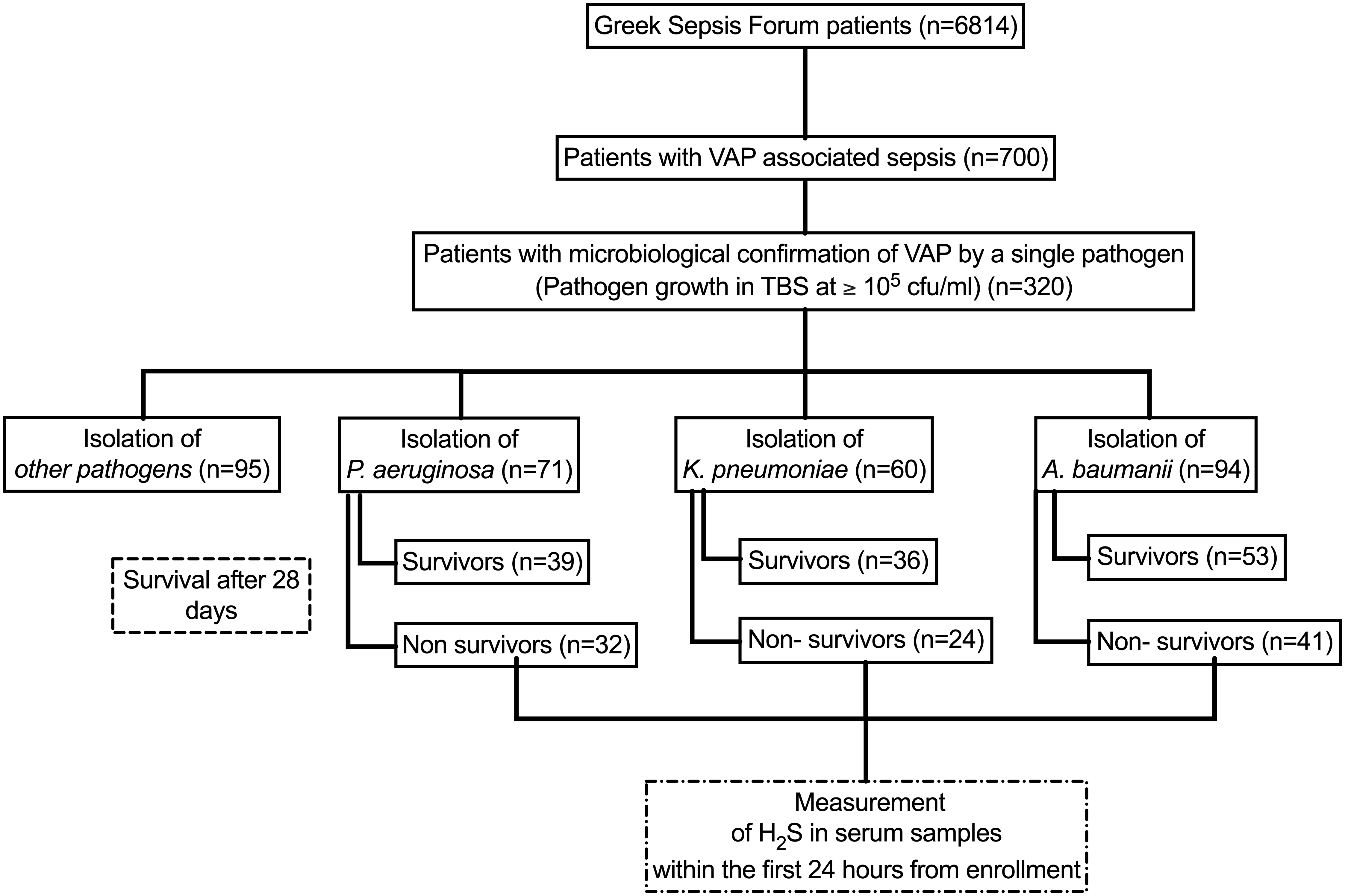

Supplement: S1 Fig — Description of the selection steps for the three groups of comparison. Abbreviations: VAP: ventilator associated pneumonia; TBS: tracheobronchial secretions. (TIF) [file ppat.1009473.s001.tif]

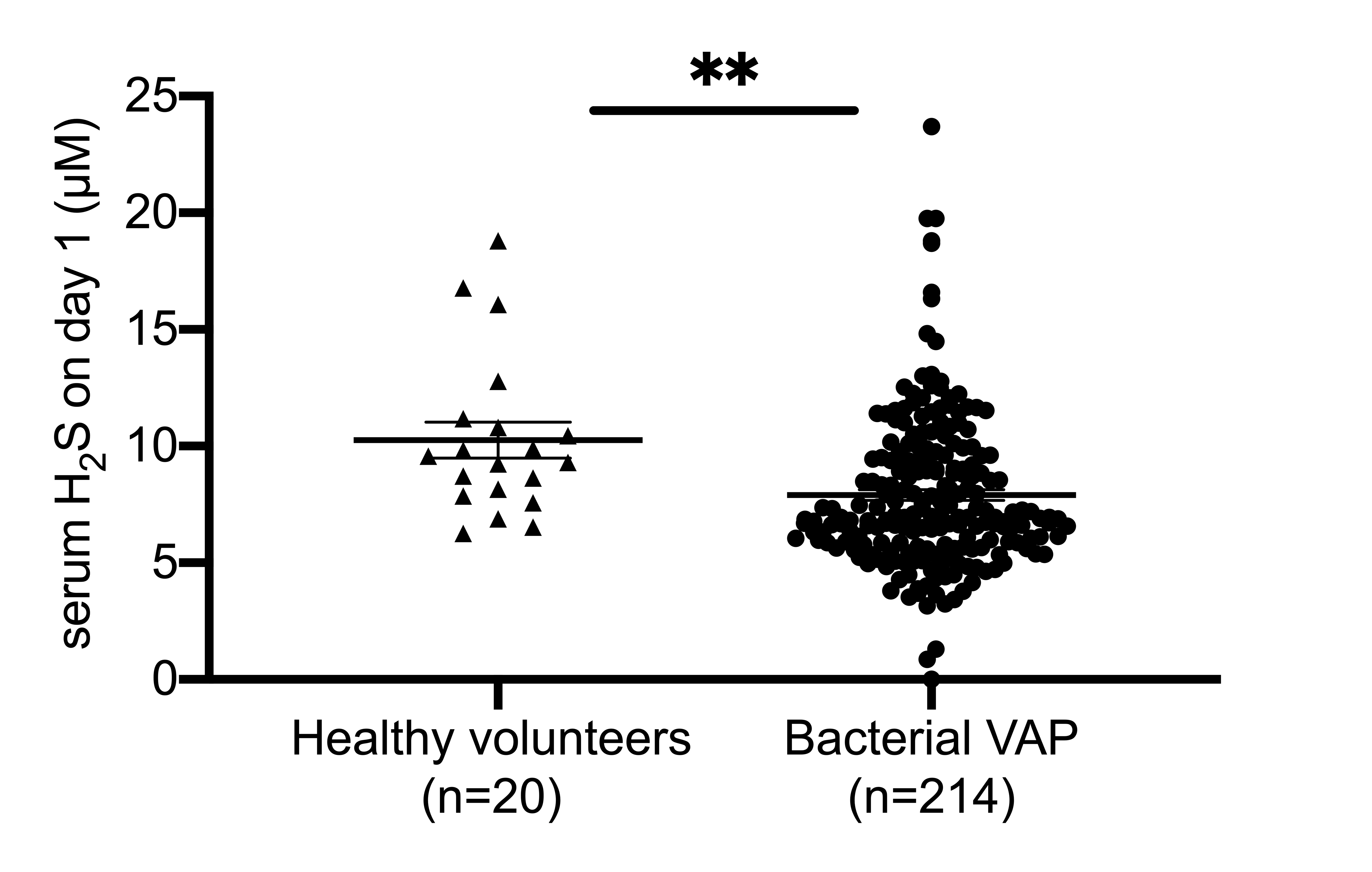

Supplement: S2 Fig — Serum levels of H2S in healthy volunteers and on day 1 among patients with sepsis due to VAP were measured by HPLC. Comparisons by the Mann Whitney U test); ✱✱ p< 0.01. (TIF) [file ppat.1009473.s002.tif]

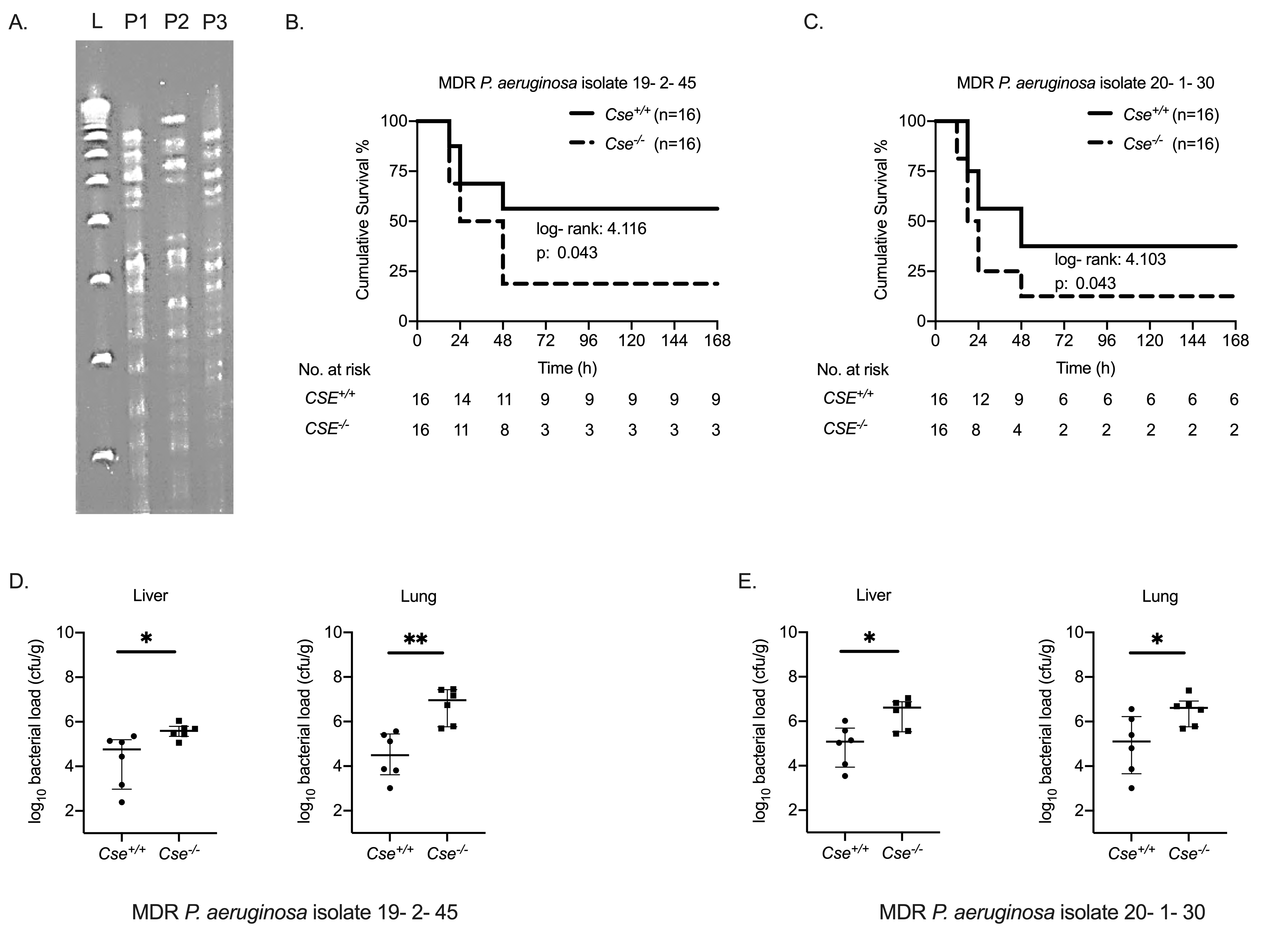

Supplement: S3 Fig — A) DNA genomic patterns of P. aeruginosa isolates 6–11–19 (P1); 19–2–45 (P2) and 20–1–30 (P3) determined by pulse field gel electrophoresis. L: DNA Ladder. Survival analysis between B) Cse+/+ and Cse-/- mice after infection with MDR P. aeruginosa isolate 19–2–45; C) Cse+/+ and Cse-/- mice after infection with MDR P. aeruginosa isolate 20–1–30; Results of the log-rank test and the relevant p- values are given. D) Bacterial load (cfu/g) in the liver and in the lung of Cse+/+ and Cse-/- mice after infection with MDR P. aeruginosa isolate 19–2–45; E) Bacterial load (cfu/g) in the liver and in the lung of Cse+/+ and Cse-/- mice after infection with MDR P. aeruginosa isolate 20–1–30. Comparisons by the Mann Whitney U test. ✱ p< 0.05, ✱✱ p< 0.01. (TIF) [file ppat.1009473.s003.tif]

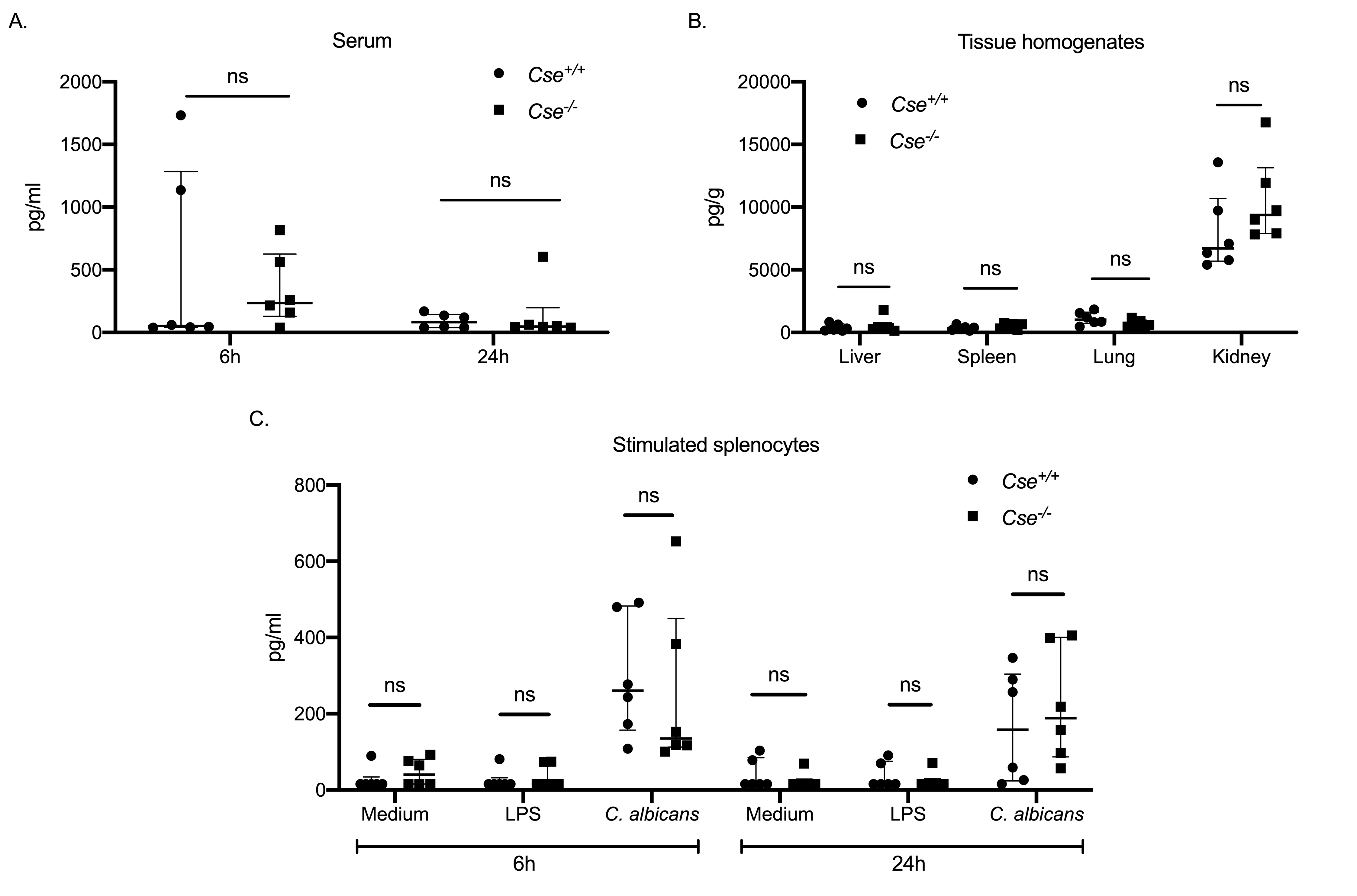

Supplement: S4 Fig — Cse+/+ and Cse-/- mice (n = 6 per group per timepoint) were sacrificed 6 and 24 hours after experimental infection by MDR P. aeruginosa isolate 6–11–19. Concentration of TNFα A) in serum, Β) tissue supernatants and C) supernatants of stimulated splenocytes (stimuli medium, LPS from Escherichia coli O55:B5 for 24h and C. albicans for 5 days). Comparison by the Mann Whitney U test; ns non- significant. (TIF) [file ppat.1009473.s004.tif]

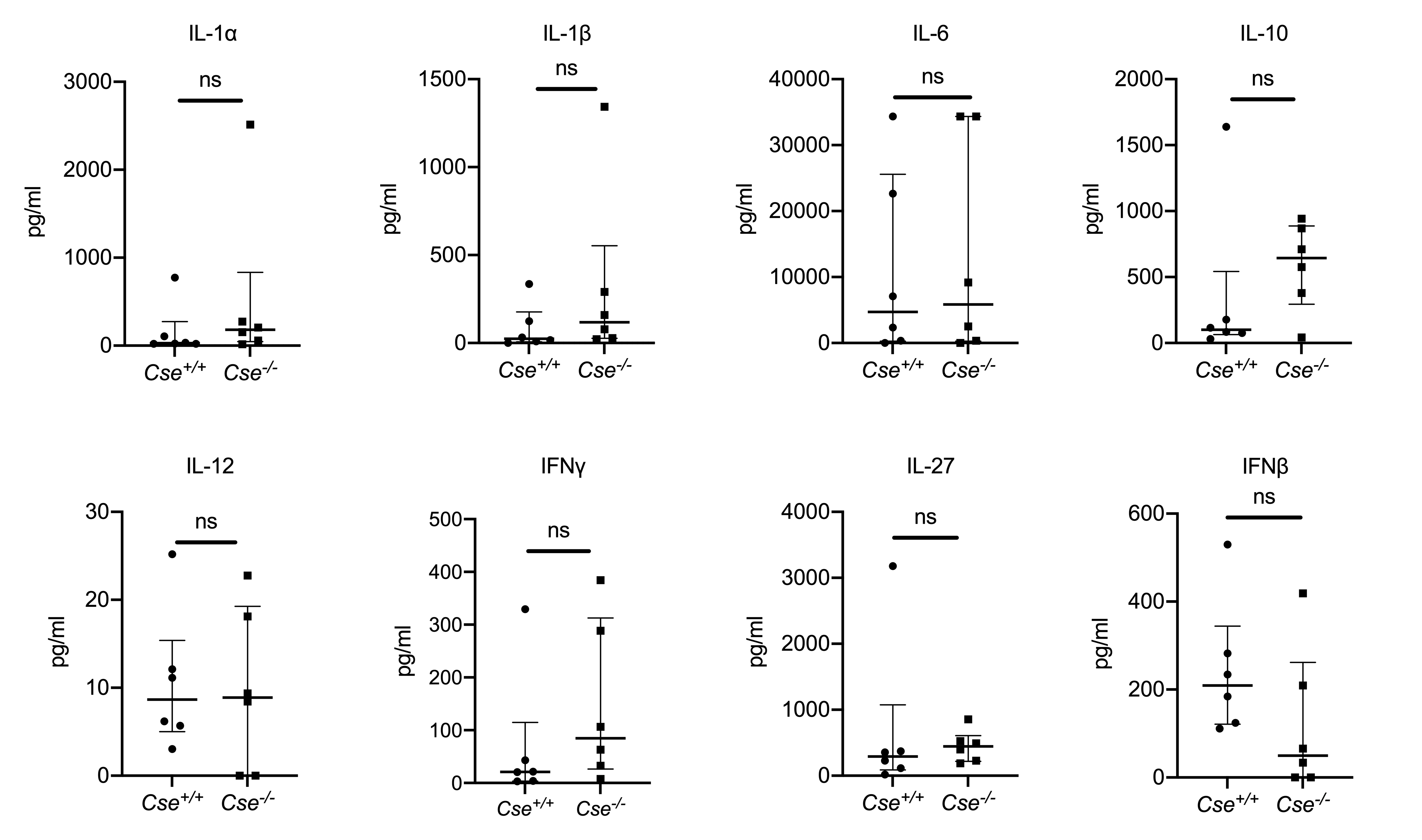

Supplement: S5 Fig — Cse+/+ and Cse-/- mice (n = 6) were sacrificed 6 hours after experimental infection by MDR P. aeruginosa isolate 6–11–19. Concentration of IL-1α, IL-1β, IL-6, IL-10, IL-12, IL-27, IFNγ, IFNβ in serum. Comparison by the Mann Whitney U test; ns non- significant. (TIF) [file ppat.1009473.s005.tif]

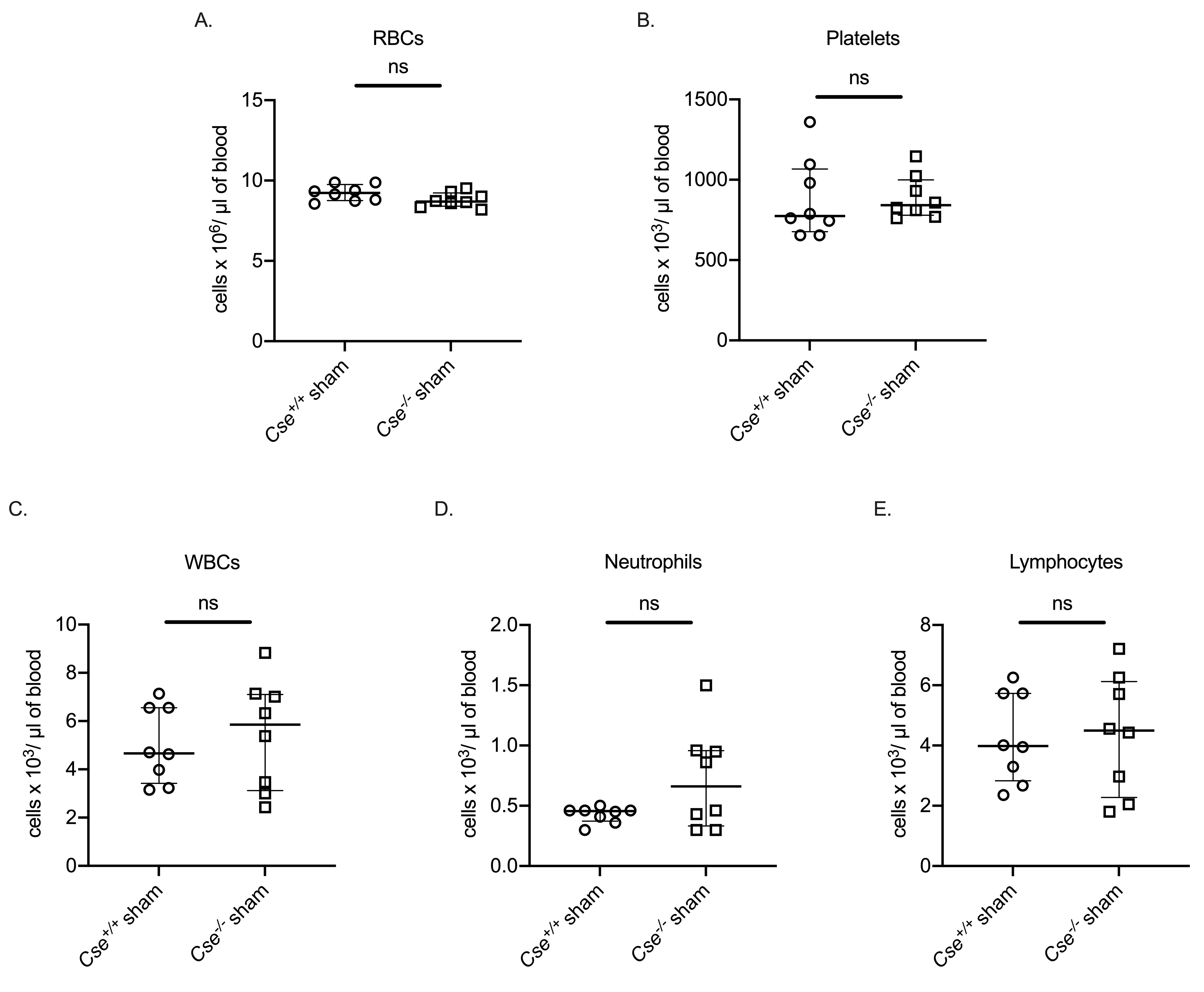

Supplement: S6 Fig — Healthy Cse+/+ and Cse-/- mice (n = 8 per group) were sacrificed. Absolute counts of A) red blood cells (RBCs); B) platelets; C) white blood cells (WBCs); D) neutrophils and E) lymphocytes were determined. Comparison by the Mann Whitney U test; ns non- significant. (TIF) [file ppat.1009473.s006.tif]

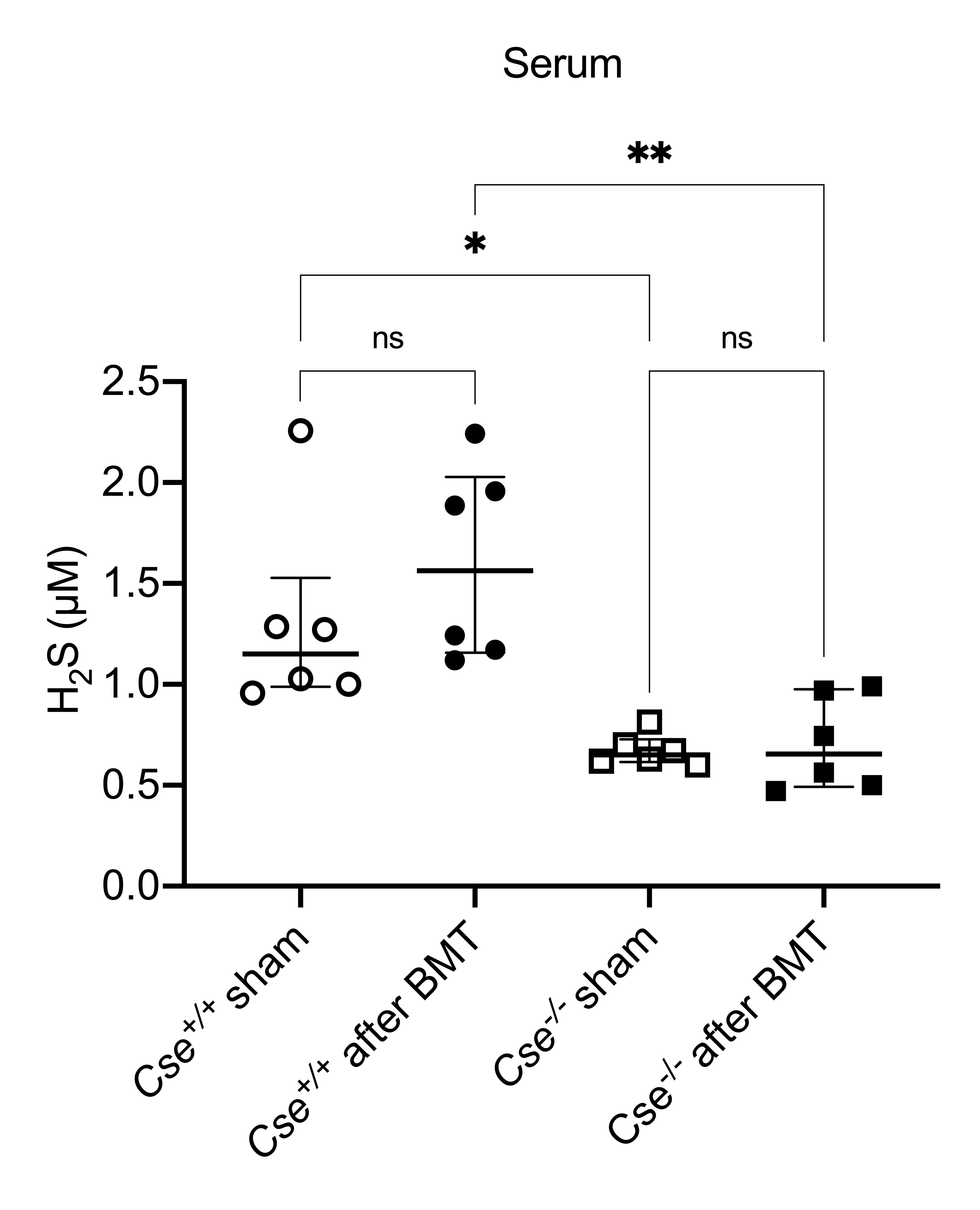

Supplement: S7 Fig — H2S levels in serum of naïve Cse+/+ and Cse-/- mice before and after bone marrow transplantation (BMT) were measured bu high- performance liquid chromatography (HPLC). Comparison by the ANOVA test with Bonferroni correction for multiple comparisons; ns non- significant, ✱ p< 0.05, ✱✱ p< 0.01. (TIF) [file ppat.1009473.s007.tif]

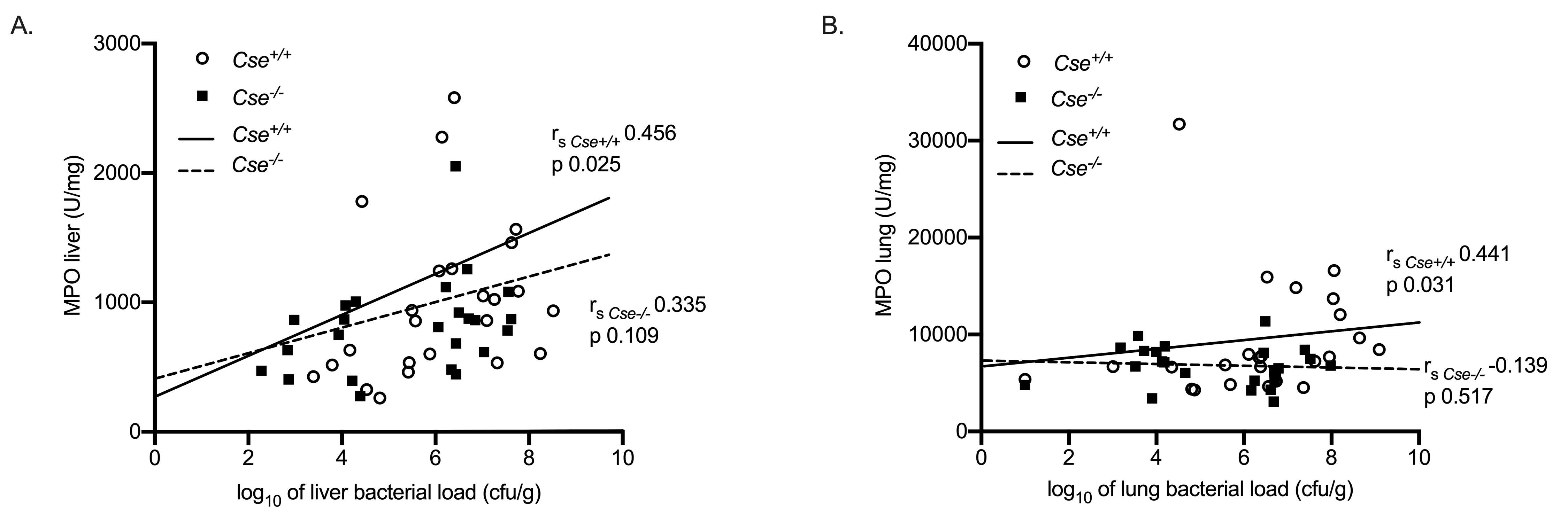

Supplement: S8 Fig — Cse+/+ and Cse-/- mice (n = 6 per group per timepoint) were sacrificed 6, 12 and 24 hours after experimental infection by MDR P. aeruginosa isolate 6–11–19. Correlations between bacterial outgrowth and MPO in the liver and in the lung for each group. Spearmann rank correlation coefficient (rs), relevant p- value and interpolation line for each group are given. (TIF) [file ppat.1009473.s008.tif]

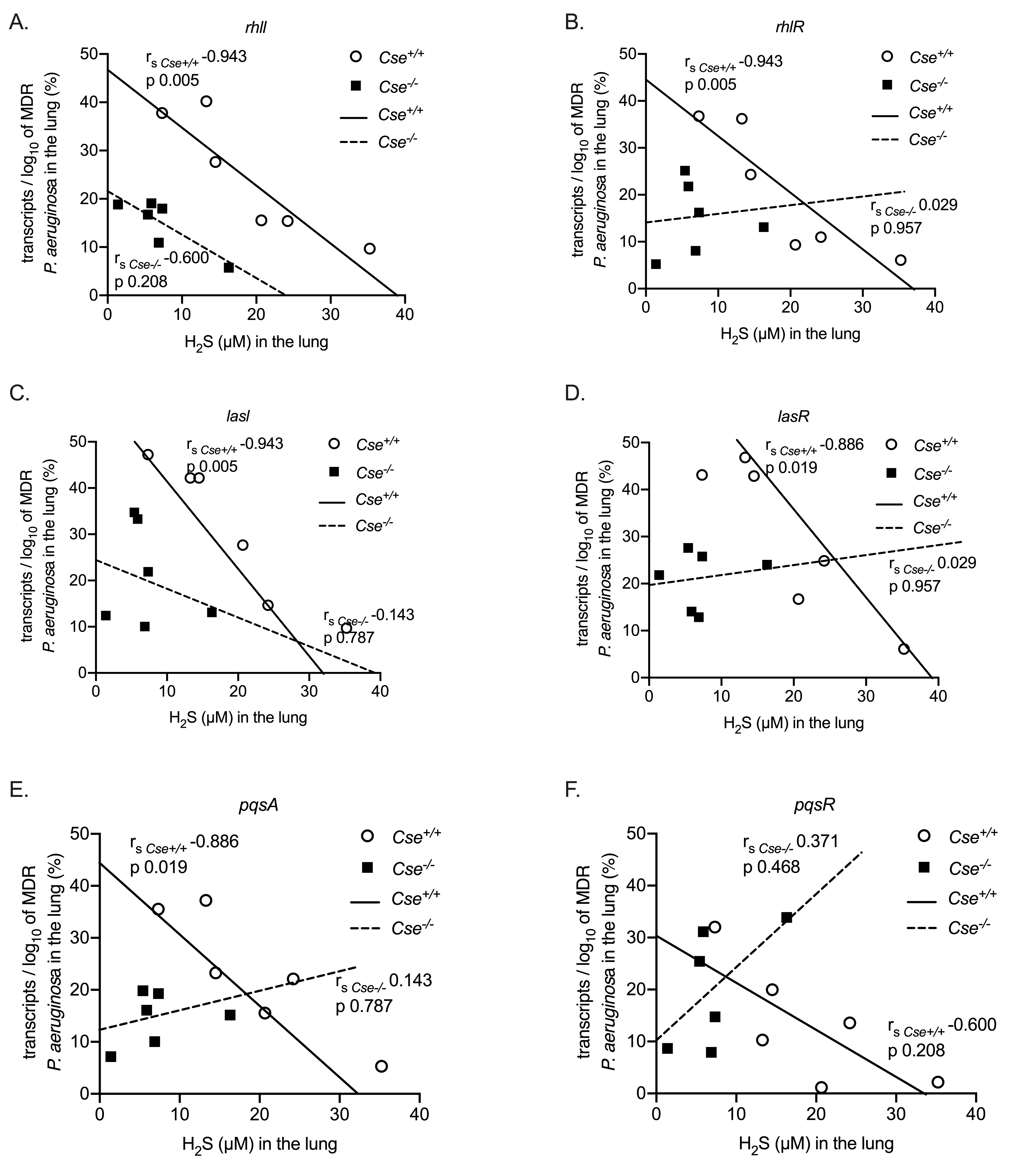

Supplement: S9 Fig — A-F) Correlation between Transcripts of QS genes rhII, rhIR, lasI, lasR, pqsA, pqsR per log10 of MDR P. aeruginosa in the lung and H2S levels in the lung in Cse+/+ and Cse-/- mice, sacrificed 6 after experimental infection by MDR P. aeruginosa isolate 6–11–19. (6 mice per group). Spearmann rank correlation coefficient (rs), interpolation line for each group and relevant p- value are given. (TIF) [file ppat.1009473.s009.tif]
