# Supplementary material for: Host cystathionine-γ lyase derived hydrogen sulfide protects against Pseudomonas aeruginosa sepsis
Source: PLoS Pathog. 2021 Mar 26;17(3):e1009473. doi: 10.1371/journal.ppat.1009473 (PMC8051778; doi:10.1371/journal.ppat.1009473)
Supplement: S1 Text — Baseline and clinical characteristics of patients with sepsis due to VAP grouped by pathogen Table B in S1 Text: Mobile phase gradient of HPLC Table C in S1 Text: Quorum sensing (QS) genes and primers of P. aeruginosa isolates used for qRT-PCR. (DOC) [file ppat.1009473.s010.doc]

**Supplementary Data**

**HOST CYSTATHIONINE-γ LYASE DERIVED HYDROGEN SULFIDE PROTECTS AGAINST *PSEUDOMONAS AERUGINOSA* SEPSIS**

**Georgios Renieris^1^, Dionysia-Eirini Droggiti^1^, Konstantina Katrini^1^,**

**Panagiotis Koufargyris^1^, Theologia Gkavogianni^1^, Eleni Karakike^1^,**

**Nikolaos Antonakos^1^, Georgia Damoraki^1^, Athanasios Karageorgos^1^,**

**Labros Sabracos^1^, Antonia Katsouda^2^, Elisa Jentho^3,4,5^, Sebastian Weis^3,4,5^,**

**Rui Wang^6^, Michael Bauer^3^, Csaba Szabo^7,8^, Kalliopi Platoni^9^,**

**Vasilios Kouloulias^9^, Andreas Papapetropoulos^2,10^,**

**Evangelos J. Giamarellos-Bourboulis^1^**

^1^4^th^ Department of Internal Medicine, National and Kapodistrian University of Athens, Medical School, Athens, Greece

^2^ Center of Clinical, Experimental Surgery & Translational Research, Biomedical Research Foundation of the Academy of Athens, Athens, Greece

^3^ Department of Anesthesiology and Intensive Care, Jena University Hospital, Jena, Germany

^4^ Institute for Infectious Disease and Infection Control, Jena University Hospital, Jena, Germany

^5^ Center for Sepsis Control and Care, Jena University Hospital, Jena, Germany

^6^ Department of Biology, York University, Toronto, Canada

^7^ Department of Anaesthesiology, University of Texas Medical Branch, Galveston, Texas, USA

^8^ Chair of Pharmacology, Department of Medicine, University of Fribourg, Fribourg, Switzerland

^9^ 2^nd^ Department of Radiology, National and Kapodistrian University of Athens, Medical School, Athens, Greece

^10^Laboratory of Pharmacology, Faculty of Pharmacy, National and Kapodistrian University of Athens, Medical School

| **Table A:** Baseline and clinical characteristics of patients with sepsis due to VAP grouped by pathogen | | | | |
| --- | --- | --- | --- | --- |
|  | *P. aeruginosa* | *K. pneumoniae* | *A. baumanni* | p value |
|  | (n=71) | (n=60) | (n=94) |  |
| Male gender (n, %) | 47 (66.2) | 36 (60.0) | 60 (63.8) | 0.764 |
| Age (mean ± SD) | 63.27 ± 17.40 | 65.78 ± 16.01 | 62.77 ± 17.49 | 0.557 |
| APACHE II (mean ± SD) | 19.69 ± 7.21 | 21.21 ± 7.48 | 18.43 ± 7.35 | 0.077 |
| CCI (mean ± SD) | 3.43 ± 2.75 | 4.17 ± 2.64 | 3.23 ± 2.38 | 0.086 |
| SOFA day 1 (mean ± SD) | 8.86 ± 3.57 | 9.15 ± 3.35 | 9.09 ± 3.55 | 0.877 |
| H_2_S in serum on day 1 (mean ± SD) | 7.85 ± 5.13 | 7.51 ± 3.31 | 7.49 ± 3.01 | 0.819 |
| Septic shock (n, %) | 47 (66.2) | 40 (66.7) | 59 (62.7) | 0.419 |
| Pathogen isolation in a blood sample (n%) | 17 (23.9) | 21 (35.0) | 14 (37.5) | 0.066 |
| Appropriateness of antimicrobial therapy (n%) | 46 (64.8) | 32 (53.3) | 62 (65.9) | 0.974 |
| Intake of corticosteroids (n%) | 11 (15.5) | 9 (15.0) | 7 (7.4) | 0.154 |
| Main comorbidities (n%) |  |  |  |  |
| ● Diabetes mellitus Typ 2 | 6 (8.5) | 5 (8.3) | 9 (9.6) | 0.114 |
| ● Chronic heart failure | 7 (9.9) | 5 (8.3) | 10 (11.0) | 0.067 |
| ● Coronary heart disease | 10 (14.3) | 10 (16.7) | 13 (14.3) | 0.100 |
| ● COPD | 6 (8.5) | 6 (10.0) | 10 (10.9) | 0.105 |
| ● Chronic renal failure | 1 (1.4) | 0 (0) | 1 (1.0) | 0.286 |
| ● Solid tumor | 2 (2.8) | 1 (1.7) | 1 (1.0) | 0.225 |
| ^A^. Abbreviations VAP: Ventilator associated pneumonia; APACHE: Acute physiology and chronic health evaluation; CCI: Charlson’s Comorbidity Index; SOFA: Sequential organ failure assessment; H_2_S: hydrogen sulfide; OR: Odds ratio; HR: Hazard ratio; CI: Confidence intervals; COPD: Chronic obstructive pulmonary disorder | | | | |
| ^B^. Comparisons between groups by one-way ANOVA test | | | | |

| **Table B:** Mobile phase gradient of HPLC | | |
| --- | --- | --- |
| Time (min) | Phase A | Phase B |
| 0 | 15 | 85 |
| 5 | 35 | 65 |
| 16 | 55 | 45 |
| 23 | 70 | 30 |
| 24 | 90 | 10 |
| 26 | 90 | 10 |
| 28 | 15 | 85 |
| ^A^. Abbreviations HPLC: High- performance liquid chromatography | | |

| **Table C:** Quorum sensing (QS) genes and primers of *P. aeruginosa* isolates used for qRT-PCR | | | |
| --- | --- | --- | --- |
| Gene | Sense primers | Antisense primers | Product size (bp) |
| rpsL^✱^ | 5΄- CCTCGTACATCGGTGGTGAAG-3΄ | 3΄- CCCTGCTTACGGTCTTTGACAC -5΄ | 148 |
| lasl | 5΄- CGCACATCTGGGAACTCA-3΄ | 3΄- CGGCACGGATCATCATCT -5΄ | 176 |
| lasR | 5΄- CTGTGGATGCTCAAGGACTAC -3΄ | 3΄- AACTGGTCTTGCCGATGG -5΄ | 133 |
| rhll | 5΄- CTGTGGATGCTCAAGGACTAC -3΄ | 3΄- CGGCATCAGGTCTTCATCG -5΄ | 101 |
| rhlR | 5΄- CTGTGGATGCTCAAGGACTAC -3΄ | 3΄- CGGTCTGCCTGAGCCATC -5΄ | 160 |
| pqsA | 5΄- CTGTGGATGCTCAAGGACTAC -3΄ | 3΄- GCTGAACCAGGGAAAGAAC -5΄ | 74 |
| PqsA | 5΄- CTGTGGATGCTCAAGGACTAC -3΄ | 3΄- ATCGACGAGGAACTGAAGA -5΄ | 142 |
| 1. Abbreviations qRT- PCR: quick real-time polymerase chain reaction 2. ^✱^ Reference/endogenous control gene | | | |
