# Supplementary material for: Host cystathionine-γ lyase derived hydrogen sulfide protects against Pseudomonas aeruginosa sepsis
Source: PLoS Pathog. 2021 Mar 26;17(3):e1009473. doi: 10.1371/journal.ppat.1009473 (PMC8051778; doi:10.1371/journal.ppat.1009473)
Supplement: Attachment — Submitted filename: PPATHOGENS-D-20-02306.docx [file ppat.1009473.s011.docx]

The manuscript by G. Renieris investigate the role of H_2_S production by CSE ininfections by *Pseudomonas aeruginosa*. First the authors show that in human serum H_2_S is lower in severe case of sepsis du to VAP by. *P. aeruginosa*. This is somehow specific to this pathogen as the benefits of H_2_S were not observed in sepsis caused by other pathogens. Importantly, in mice, data show that mortality is higher in mice lacking CSE (whole body KO), and could be restored by exogenous administration of H_2_S via STS or GYY. It is a welcome choice to use STS as this drug is clinically approved, although its role as an H_2_S donor still need to be clearly demonstrated. Next, the authors investigate the mechanisms that lead to death in the case of host H_2_S deficiency. First, they show that in mice rendered neutropenic by cyclophosphamide, the survival benefit of CSE was lost. Next, they observed increase bacterial load in CSE KO mice, attributed to decrease phagocytic capacity. Finally, the authors showed that the specific susceptibility of CSE KO mice to *P.aeruginosa* infection was due to an H_2_S dependent down-regulation of quorum sensing genes of  *P.aeruginosa.*

Taken together, this is an **interesting study** that addressed the role H_2_S in P. aeruginosa and sepsis. This represent **novel findings**, with potentially important clinical implication (as stated by the authors *“*Pseudomonas (P.) aeruginosa are responsible for more than 50% of VAP cases in Greece and are characterized by high rates of antimicrobial resistance”). While some of the data are very convincing, there are some areas that **require additional experiments** to address the key questions and to potentially support the conclusions. This would also be needed **before it is suited for publication in this journal**. As the neutrophil seem to be critical cellular mediator of the phenotype in CSE KO mice, and since CSE is a promiscuous enzyme, bonne marrow transfer (or CSE removal in neutrophil specifically) would be valuable. I am also wondering what the impact on serum H_2_S in this setting would be. Next, and from a clinical perspective it would be interesting to see the effect of H_2_S administration in WT mice (survival after *P.aeruginosa* exposure). In line with this, I would be curious to see if H_2_S administration after the infection could rescue the mice. Finally, it remains unclear by which mechanism H_2_S targets quorum sensing genes of *P.aeruginosa* (could it be linked to linked sulfhydration of NF-κB**,** PMID: 22244329). The following points/questions should also be address in more detail:

1. Figure 1 and methods: precise where and how the heathy serum was obtained. What are their baseline comorbidities (if any)? Are they matched for anything (e.g. age )?

2. Figure 1A. It appears that the H_2_S levels in the survivors’ group from P. aeruginosa infection are significantly higher that the others. Can you provide some reasonable explanation?

3. Could you pool the data from the sepsis group, which would indicate lower H_2_S levels in patients with sepsis compare to healthy patients? This is somehow counterintuitive as H_2_S seems to increase upon infection in mice (Figure 3)

4. Figure S1. The Yes / No box significance isn’t clear.

5. Figure 1B. Could you provide the H_2_S levels value distribution for all of the sample? How do you explain that H_2_S values between studies, using the same methods (PMID: 32433216) differs greatly (x100 fold difference)? I believe that threshold would therefore be difficult to apply in the clinical setting. I also believe that H_2_S could be sensitive at predicting survival, but it is hard to believe that it would be specific since it has pleiotropic role. Also other score might be more specific (e.g. SOFA).

6. Add the # at risk to every Kaplan Meier data.

7. How do you explain that survival is different in CSE KO mice from Fig 2A and B. Again provide the number at risk. Can you explain why there is some mice that seems to be long term survivors?

8. The model used here seems to be very severe as 50% of WT mice are dead at 24hrs. This is largely above what’s observed in human. This is even more concerning in Figure 3F using the LPS model. Can it be that CSE provides some “unspecific survival benefits”. The importance of CSE in longevity and stress resistance was demonstrated in several studies (e.g. Hine et al, Cell 2015).

9. Fig 3. CSE KO have significant amount of baseline H_2_S levels. Also H_2_S seems to increase upon infection also in the KO too. Is this increase significant ? I would suggest to change the X axis, and group the data by genotype (and do so in **all of the graph**).

10. Figure 3D. Please provide serum H_2_S levels in 3MST WT and KO.

11. Please provide cytokine value at baseline and not only after infection. This is of particular interest in CSE WT and KO.

Minor points

- The abstract should be revised to be more easily read and clearer.
- Spell H_2_S and not H2S.
- Figure 4E legends: Ratio of CD11b+CD11cCD45 cells to CD11b+CD11cCD45 cells doesn’t make sense.
