# Supplementary material for: Host cystathionine-γ lyase derived hydrogen sulfide protects against Pseudomonas aeruginosa sepsis
Source: PLoS Pathog. 2021 Mar 26;17(3):e1009473. doi: 10.1371/journal.ppat.1009473 (PMC8051778; doi:10.1371/journal.ppat.1009473)
Supplement: Attachment — Submitted filename: Response to reviewers.docx [file ppat.1009473.s012.docx]

**Response to reviewers**

**Part I - Summary**
Please use this section to discuss strengths/weaknesses of study, novelty/significance, general execution and scholarship.

Reviewer #1: This is an interesting study investigating the role of H2S in sepsis. This pathway has not been previously investigated in these clinical models of disease and in patients with sepsis, and the results are novel and interesting. The combination between experimental and clinical data is particularly compelling, arguing for the importance of the findings.

Reply: We would like to thank the reviewer for his positive feedback and suggestions. We have tried to address his comments and hope that the corrected version of the manuscript meets this reviewer’s criteria for publication.

Reviewer #2: The manuscript by G. Renieris investigate the role of H2S production by CSE infections by Pseudomonas aeruginosa. First the authors show that in human serum H2S is lower in severe case of sepsis du to VAP by. P. aeruginosa. This is somehow specific to this pathogen as the benefits of H2S were not observed in sepsis caused by other pathogens. Importantly, in mice, data show that mortality is higher in mice lacking CSE (whole body KO), and could be restored by exogenous administration of H2S via STS or GYY. It is a welcome choice to use STS as this drug is clinically approved, although its role as an H2S donor still need to be clearly demonstrated. Next, the authors investigate the mechanisms that lead to death in the case of host H2S deficiency. First, they show that in mice rendered neutropenic by cyclophosphamide, the survival benefit of CSE was lost. Next, they observed increase bacterial load in CSE KO mice, attributed to decrease phagocytic capacity. Finally, the authors showed that the specific susceptibility of CSE KO mice to P.aeruginosa infection was due to an H2S dependent down-regulation of quorum sensing genes of P.aeruginosa.
Taken together, this is an interesting study that addressed the role H2S in P. aeruginosa and sepsis. This represent novel findings, with potentially important clinical implication (as stated by the authors “Pseudomonas (P.) aeruginosa are responsible for more than 50% of VAP cases in Greece and are characterized by high rates of antimicrobial resistance”). While some of the data are very convincing, there are some areas that require additional experiments to address the key questions and to potentially support the conclusions. This would also be needed before it is suited for publication in this journal. As the neutrophil seem to be critical cellular mediator of the phenotype in CSE KO mice, and since CSE is a promiscuous enzyme, bonne marrow transfer (or CSE removal in neutrophil specifically) would be valuable. I am also wondering what the impact on serum H2S in this setting would be. Next, and from a clinical perspective it would be interesting to see the effect of H2S administration in WT mice (survival after P.aeruginosa exposure). In line with this, I would be curious to see if H2S administration after the infection could rescue the mice. Finally, it remains unclear by which mechanism H2S targets quorum sensing genes of P.aeruginosa (could it be linked to linked sulfhydration of NF-κB, PMID: 22244329).

Reply: We would like to thank the reviewer for his positive feedback and suggestions, especially for the recognition of the potential use of the findings in the clinical setting. We have tried to address his comments and hope that the corrected version of the manuscript meets this reviewer’s criteria for publication. The three main suggestions included in the summary are addressed separately in the following sections.

**Part II – Major Issues: Key Experiments Required for Acceptance**

Reviewer #1: Major comments

- The quality of the figures is poor, that should be improved.

Reply: We have revised the figures in order to improve their quality

- The bacterial load in the Cse^+/+^ and Cse^-/-^ mice after Pseudomonas infection is given in Fig.4F at the spleen level. Were bacterial burdens assessed also in other organs more likely to be important for determining mortality?

Reply: We have assessed the bacterial load in the liver, lung and spleen of *CSE^+/+^* and *CSE^-/-^* mice after Pseudomonas infection. Figure 4 has been revised to better depict the higher bacterial loads found in the liver, lung and spleen of *CSE^-/-^* 6 hours after infection (Figure 4C).

Reviewer #2: Major comments

- As the neutrophil seem to be critical cellular mediator of the phenotype in CSE KO mice, and since CSE is a promiscuous enzyme, bonne marrow transfer (or CSE removal in neutrophil specifically) would be valuable. I am also wondering what the impact on serum H2S in this setting would be.

Reply: We would like to thank the reviewer for his suggestion. Indeed, according to the results of this study the effect of H_2_S on neutrophils seems to be fundamental for the proposed mechanism of H_2_S mediated protection against P. aeruginosa infections. We gladly followed the advice of this reviewer and proceeded with survival and sacrifice experiments in *Cse^-/-^* mice, after bone marrow cell (BMCs) transfer from *Cse^+/+^* mice.

Firstly, we determined counts of blood cell populations in healthy Cse^+/+^ and Cse^-/-^ mice, demonstrating that there are no significant differences. In a second step, Cse^+/+^ and *Cse^-/-^* mice were irradiated (total body irradiation). 24 hours later 1x10^6^ BMCs from healthy *Cse^+/+^* mice were injected intravenously. 30 days after BM transfer, which according to the literature is sufficient for full recovery of the mice, mice were infected with the same inoculum of the MDR *P. aeruginosa* 6-11-19 isolate. Survival was recorded for 7 days. H_2_S levels were measured in serum of recipient mice before infection.

BMT did not significantly alter the H_2_S plasma levels of recipient Cse^+/+^ and Cse^-/-^ mice (Figure S6). However, BMT from a *Cse ^+/+^* donor significantly reduced the mortality of recipient *Cse ^-/-^* mice. Their mortality was similar to the survival of naïve *Cse ^+/+^* mice and *Cse ^+/+^* mice after BMT. These finding, added to our prior result prove that there is a deficit of neutrophilic activity and recruitment in *Cse ^-/-^* mice, rendering them susceptible to infection by MDR *P. aeruginosa*.

Relevant data has been added in the Materials and Methods section ln. 296-310; the Results section ln. 550-563 and the Discussion ln. 680-682.

- Next, and from a clinical perspective it would be interesting to see the effect of H_2_S administration in WT mice (survival after P. aeruginosa exposure). In line with this, I would be curious to see if H2S administration after the infection could rescue the mice.

Reply: As the reviewer mentions in his summary sodium thiosulfate (STS) is a clinically approved H_2_S donor, with however unclear effect in infection models. We have repeated the survival experiments in *Cse^+/+^* mice after Pseudomonas infection and treatment with STS. STS led to a prolongation of the survival of *Cse^+/+^* compared to untreated *Cse^+/+^* mice. Related data has been added in the Results section lines 490- 491 “Treatment with STS in *Cse ^+/+^* mice led to prolongation of survival (64.7% compared to 47.1%)”. The data is shown in Figure 1C.

- Finally, it remains unclear by which mechanism H2S targets quorum sensing genes of P.aeruginosa (could it be linked to linked sulfhydration of NF-κB, PMID: 22244329).

Reply: The exact mechanism by which hydrogen sulfide interacts with the quorum sensing system of *P. aeruginosa* remains unclear. Published data highlight the use of garlic extract, which contains several polysulfides, which in vivo stimulate the synthesis of hydrogen sulfide, as a potential quorum sensing inhibitor. Additionally, H_2_S produced by CSE has been found to up- regulate the expression and actions of NF-κΒ, whereas AHLs have been shown to down- regulate NF-κΒ pathway, which in term leads to a deficient innate immune response. Thus, it may be assumed that it is the up-regulation of the NF-κΒ pathway mediated by H_2_S, which is responsible for the limitation of the activation of the quorum sensing system of *P. aeruginosa*.

The relevant section has been added in the manuscript ln. which now reads: “Previous studies show that garlic extract, containing several polysulfides, which in vivo stimulate the synthesis of hydrogen sulfide, inhibits the quorum sensing system of *P. aeruginosa* rendering it vulnerable to elimination through polymorphonuclear cells [40–42]. However, the exact mechanism by which H_2_S regulates the expression of the quorum sensing genes of P. aeruginosa still remains unclear. AHLs have been shown to reduce the activation of the nuclear factor-kappa B (NF-κΒ) pathway, which in term leads to a deficient innate immune response [43,44]. On the contrary, H_2_S produced by CSE has been found to up- regulate the expression and actions of NF-κΒ, through sulfhydration [45]. Thus, the up-regulation of the NF-κΒ pathway mediated by H_2_S is a potential mechanism for the limitation of the activation of the quorum sensing system of *P. aeruginosa.”* Relevant data has been added in the manuscript ln. 736-746.

The following points/questions should also be address in more detail:

- Figure 1 and methods: precise where and how the heathy serum was obtained. What are their baseline comorbidities (if any)? Are they matched for anything (e.g. age )?

Reply: Blood samples were also isolated from healthy volunteers. The volunteers had no comorbidities, as the ones depicted in table 1 and were selected to match the patients with VAP for age and gender. The relevant addition has been made in the manuscript ln. 156-157.

- Figure 1A. It appears that the H2S levels in the survivors’ group from P. aeruginosa infection are significantly higher that the others. Can you provide some reasonable explanation?

Reply:

As it has been demonstrated in the in vivo experiments, higher H_2_S levels in serum are related to an increased survival through a more efficient elimination of *P. aeruginosa* and also through inhibition of the quorum sensing system of *P. aeruginosa*. In patients serum H_2_S levels of survivors from *P. aeruginosa* infection were significantly higher than H_2_S levels of non- survivors. However, no significant differences were found compared to healthy volunteers as well as survivors from infection by *A. baumanni* or *K. pneumoniae*. Moreover, serum H_2_S levels of non- survivors from *P. aeruginosa* infection were decreased compared to healthy volunteers. Thus, we believe that defective host- derived H_2_S responses in septic patients enables the increased expression of the quorum sensing system of *P. aeruginosa* infections leading to raised bacterial loads and subsequent raised mortality.

- Could you pool the data from the sepsis group, which would indicate lower H2S levels in patients with sepsis compare to healthy patients? This is somehow counterintuitive as H2S seems to increase upon infection in mice (Figure 3)

Reply: Pooling all the septic patients together it results that H2S serum levels of septic patients were significantly lower compared to healthy comparators. Data has been added in the manuscrript lns. 440-441 and Fig. S2. However, interpreting our results, it is clear that patients who sustained serum H_2_S levels similar to the H_2_S levels of healthy volunteers, had a significantly lower risk of an unfavorable outcome compared to septic patients who failed to sustain H_2_S levels. This is in accordance with the in vivo data, where H_2_S supplementation by STS administration increased survival in *CSE^-/-^* but not in *CSE^+/+^* mice. Overall, we believe that it is the H_2_S deficiency that correlates with elevated mortality.

- Figure S1. The Yes / No box significance isn’t clear.

Reply: The figure has been changed to depict the number of survivors and non- survivors in each group at day 28 after enrolment.

- Figure 1B. Could you provide the H2S levels value distribution for all of the sample? How do you explain that H2S values between studies, using the same methods (PMID: 32433216) differs greatly (x100 fold difference)? I believe that threshold would therefore be difficult to apply in the clinical setting. I also believe that H2S could be sensitive at predicting survival, but it is hard to believe that it would be specific since it has pleiotropic role. Also other score might be more specific (e.g. SOFA).

Reply: Our data verify the comment of the reviewer. Indeed, as depicted in figure 1 sustained serum H_2_S levels is characterised by a great sensitivity for predicting survival but also by a mediocre specificity. Therefore, we believe that serum H_2_S could be utilized as an adjunctive criterion, together with other established severity scores such as SOFA, which characteristically is another independent prognostic factor for unfavorable outcome in our cohort (Table 1), and other biomarkers such as CRP and PCT, for decision making. The relevant addition has been made in manuscript ln. 778-780.

Concerning, the first part of the comment, the median of the all of the study sample being 7.25μM with a standard deviation of 3.38, which was significantly lower compared to the values measured in the patients with COVID-19. However, as it is pointed out in the study which is referenced by the reviewer and was performed from our research group (PMID: 32433216), we believe that elevated H_2_S levels in serum of COVID-19 patients are a protective mechanism for an unfavourable outcome. Thus, we believe that H_2_S increase may well be an intrinsic characteristic of Covid-19 described for the first time in the referenced paper. Moreover, previous studies measuring H_2_S plasma levels in humans show a great variance. H_2_S levels seem to be disease- and pathogen specific and also great influenced by the measuring method. Thus, this great variance makes the application of a H_2_S threshold difficult. H_2_S levels as a biomarker have to be used individually for separate pathogens. Recognizing this limitation, the relevant addition has been made in the manuscript which now reads in ln. 768-774 as follows “The translation into the clinical setting of findings of pre-clinical studies on the role of H_2_S in infection poses a great challenge, mainly because of the great variance of measured H_2_S levels in the plasma, which ranges from 2μM to over 500μM [6,51–53]. The measured levels seem to be disease- and pathogen- specific and also depend on the measuring method. This variance renders the use of a general threshold of H_2_S plasma levels difficult and necessitates a more individual approach.”

- Add the # at risk to every Kaplan Meier data.

Reply: The number at risk has been added to every Kaplan Meier curve as suggested.

- How do you explain that survival is different in CSE KO mice from Fig 2A and B. Again provide the number at risk. Can you explain why there is some mice that seems to be long term survivors?

Reply: The survival of *Cse^-/-^* mice in the two diagrams are equal. The presentation of the groups was probably misleading, because the same style of reference line was used for different groups. Figures have been revised. The number at risk has been added. Concerning the long-term surviving mice, it seems that the protective effect of H_2_S produced by CSE occurs in the first 72 hours after experimental infection, leading to a more effective pathogen eradication during this time-period. Pathogen eradication seems to be the explanation for long term survivors of all groups.

- The model used here seems to be very severe as 50% of WT mice are dead at 24hrs. This is largely above what’s observed in human. This is even more concerning in Figure 3F using the LPS model. Can it be that CSE provides some “unspecific survival benefits”. The importance of CSE in longevity and stress resistance was demonstrated in several studies (e.g. Hine et al, Cell 2015).

Reply: Intention of this study was to evaluate the role of hydrogen sulfide in a lethal infection model. Therefore, we used inocula of the selected pathogens, which have been shown to cause an infection with high mortality. The inoculum 1 x 10^8^ cfu/ml MDR *P. aeruginosa* which was used, has been shown to cause an up to 60% mortality in C57bl/6 mice, as cited in the manuscript. Most of the mice that succumb to the infection, do so within the first 24 hours. This is the reason, why the complete study design focuses on these first hours after infection (6h sacrifices), which seem to be crucial for the survival of the infected mice. Of course, we do agree with the comment of the reviewer, which is supported by various studies, that CSE derived H_2_S affords protection against various forms of stress. The relevant addition has been made in the manuscript, which now reads in ln. 666-672 as follows “Hydrogen sulfide production through the CSE enzyme has been observed both in pro- and eucaryotic organisms. This evolutionary conservation of CSE derived H_2_S, together with findings from several studies, which have demonstrated H_2_S as a mediator of several human physiological functions, longevity and stress resistance, have shifted the point of view of the research community away from considering H_2_S as a toxic gas and recognizing its place as a third gaseous transmitter”.

Concerning the increased mortality after LPS challenge in both groups, the finding is consistent with previously published data (Ahmad et al. PMID: 27748832), which have shown that the survival rates after 20mg/kg LPS challenge led to a 10% mortality in WT and Cse-/- mice within 40 hours after challenge. This is referenced in the manuscript in ln. 536- 539.

- Fig 3. CSE KO have significant amount of baseline H2S levels. Also H2S seems to increase upon infection also in the KO too. Is this increase significant ? I would suggest to change the X axis, and group the data by genotype (and do so in all of the graph).

Reply: We have made the change requested by the reviewer concerning the related graphs. As described in the manuscript H_2_S production is mediated by three different enzymatic systems, cystathionine γ- lyase (CSE), cystathionine β- synthase (CBS), and 3-mercaptopyruvate sulfurtransferase (3MST). H_2_S is present in CSE and 3MST KO mice, as its production is supported by the existing enzymes. The increase found in infected was not statistically significant and the relevant addition has been made in the manuscript ln. 514-515 which reads as follows: “Moreover, H_2_S levels in serum increased after infection by MDR *P. aeruginosa*, but only in *Cse^+/+^* mice.”

- Figure 3D. Please provide serum H2S levels in 3MST WT and KO.

Reply: According to the results of this study, serum H_2_S is a key factor for survival after P. aeruginosa infection. It has been shown that 3*Mst* deficiency does not significantly alter serum H_2_S levels in mice (reference 28). This is in accordance with our finding that survival after P. aeruginosa infection does not differ significantly between *3Mst^+/+^* and *3Mst^-/-^*. This finding supports the hypothesis that CSE derived H_2_S is responsible for protection against *P. aeruginosa* infection.

The manuscript now reads in lines 515-522 as follows: “In order to study the importance of the enzymatic system of host H_2_S production, we performed another set of experiments using mice with homozygous deficiency for *3Mst*. *3Mst* deletion does not lead to a significant alteration of serum H_2_S levels in mice [28]. Indeed, survival from infection by MDR *P. aeruginosa* was similar between *3Mst^+/+^* (42.9%) *3Mst^-/-^* (38.5%) mice (Fig 3D). These findings demonstrate that the susceptibility of the host for infection by *P. aeruginosa* is selectively driven by H_2_S produced through the catalytic activity of CSE.”

- Please provide cytokine value at baseline and not only after infection. This is of particular interest in CSE WT and KO.

Reply: According to the reviewer’s suggestion, we measured serum cytokines in non- infected *CSE^+/+^* and *CSE^-/-^* mice. MCP-1, IL-23 and GM-CSF, differed between infected *CSE^+/+^* and *CSE^-/-^* mice, but not between non- infected mice of the two groups. Moreover, a significant increase of these cytokines following infection was demonstrated but only in *CSE^+/+^* mice. Thus, it can be concluded that CSE derived H2S regulates the expression of these inflammatory mediators which mediate neutrophil chemotaxis and activity.

The manuscript now reads in lines 565-566 as follows: “Moreover, the expression of these mediators increased after infection but only in *Cse ^+/+^* mice.”

**Part III – Minor Issues: Editorial and Data Presentation Modifications**

Reviewer #1: Minor suggestions

- There is probably a mistake in the sentence: “The ratio of CD11b+CD11c-CD45 to CD11b+CD11c-CD45 of the spleen, which mostly represent the ratio of the granulocyte to the macrophage population [28]”, as I guess different cell markers need to be used for the two populations.

Reply: The relevant part has been corrected and now reads “Ratio of CD11b^+^CD11c^-^CD45 cells to CD11b^-^CD11c^+^CD45”.

- The authors conclude based on the circulating cytokine measurements that “the protective role is not associated with an effect on cytokine production by monocytes or lymphocytes”. However, one paragraph later they show decreased production of mediators produced by neutrophils. It is indeed cytokine production locally which is more important, rather than circulating cytokine concentration in the blood, so I would suggest to change this conclusion.

Reply: We agree with the comment of the reviewer. The statement has been changed and reads in lines 547-548 as follows: “This suggests that the protective role is not directly associated with a change in the concentration in the blood of circulating cytokines.”

- Can the authors speculate about the functionality of the H2S system in patients with cystic fibrosis?

Reply: We would like to thank the reviewer for his comment. Data about the function of hydrogen sulfide in patients with cystic fibrosis is lacking. Cowley et al. analyzed sputum samples from 22 children with CF showed that higher concentrations of H_2_S in sputum were inversely correlated with the amount of sputum produced and was negatively correlated with the likelihood for inpatient hospitalization or oxygen supplementation, suggesting that sulfide could be a marker of good health in CF patients. This effect of hydrogen sulfide was attributed to an enhancement of the reduction capacity, possibly rendering the airway microenvironment inhospitable for aerobes. This data has been added in the discussion ln. 720-726.

Reviewer #2:

- The abstract should be revised to be more easily read and clearer.

Reply: The abstract has been revised as suggested by the reviewer.

- Spell H2S and not H2S.

Reply: The term has been corrected in the whole of the manuscript.

- Figure 4E legends: Ratio of CD11b+CD11cCD45 cells to CD11b+CD11cCD45 cells doesn’t make sense.

Reply: The relevant part has been corrected and now reads “Ratio of CD11b^+^CD11c^-^CD45 cells to CD11b^-^CD11c^+^CD45”.
